# Effects of the eccentric chin closure exercise on submental muscle activation, muscle strength, dysphagia limit, perceived exertion and pain in healthy volunteers: A prospective, randomized parallel group study

**DOI:** 10.1371/journal.pone.0313995

**Published:** 2024-11-21

**Authors:** Emre Cengiz, Selen Serel Arslan, Ömer Faruk Yaşaroğlu, Rabia Alıcı, Numan Demir, Mehmet Akif Topçuoğlu, Akmer Mutlu

**Affiliations:** 1 Hacettepe University, Graduate School of Health Sciences, Ankara, Turkey; 2 Faculty of Health Sciences, Department of Physiotherapy and Rehabilitation, Uşak University, Uşak, Turkey; 3 Faculty of Physical Therapy and Rehabilitation, Hacettepe University, Ankara, Turkey; 4 Department of Neurology, Hacettepe University Hospitals, Ankara, Turkey; Nishikyushu University: Nishikyushu Daigaku, JAPAN

## Abstract

**Objective(s):**

Eccentric Chin Closure (ECC) exercise is a model designed to strengthen the suprahyoid muscles, aligned with the principles of eccentric exercise and the characteristics of these muscles. This study aimed to investigate the effects of the ECC exercise on submental muscle activation, muscle strength, dysphagia limit, perceived exertion, and pain, in comparison to the Shaker and Chin-Tuck Against Resistance (CTAR) exercises.

**Methods:**

**In this parallel randomized controlled trial,** for the initial assessment fifty-four healthy volunteers aged between 19–28 years with submental activations were recorded during the isotonic components of the Shaker, CTAR, and ECC exercises using surface electromyography. After the initial assessment, the volunteers were randomized to the Shaker, CTAR, and ECC exercise groups with 18 volunteers each group, and followed an 8-week exercise program. Maximum voluntary isometric contractions (MVC), muscle strength, dysphagia limit, perceived exertion, and pain were recorded at baseline in 4^th^ week and 8^th^ week.

**Results:**

At the initial assessment, lower submental muscle activation was observed during the Shaker exercise (*p<*0.05). Follow-up measurements demonstrated that the eight weeks of exercise was effective in increasing MVC activations and muscle strength across all groups. Considering the group*time effect, CTAR (0.36 ± 0.10) and ECC (0.40 ± 0.14) exercises were found to be more effective in increasing MVC than the Shaker (0.29 ± 0.19) exercise (F = 7.203, p<0.001), and the ECC (32.87 ± 6.55) exercise was more effective in improving muscle strength than both the Shaker (26.03 ± 5.86) and CTAR (27.95 ± 6.33) exercises (F = 6.786, p<0.001). Perceived exertion (F = 1.044, p = 0.388) and pain scores (F = 0.346, p = 0.846) showed statistically similar changes across the Shaker, CTAR, and ECC exercise groups.

**Conclusion:**

The ECC exercise demonstrated similar effects on MVC to CTAR, but resulted in greater MVC than the Shaker exercise among healthy volunteers at 8 weeks. ECC was also more effective compared to Shaker and CTAR in terms of strength gain, with all exercises showing comparable levels of perceived exertion and pain.

## Introduction

Swallowing is a function consisting of oral preparation and oral, pharyngeal, and esophageal phases. Dysphagia defines problems in any of these phases of swallowing function [1]. Although different problems may be observed in each phase, one of the most important problems in the pharyngeal phase is insufficient airway closure, which can lead to residue and/or aspiration [2]. Hyolaryngeal elevation is one of the main movements that provides airway closure, which is performed mostly by suprahyoid muscle contractions [2,3]. Suprahyoid muscle function plays a critical role in the extent and effectiveness of hyolaryngeal elevation [4].

The principle of specific adaptation to imposed demands states that body functions change and adapt in line with expected specific tasks [5]. While the exercise is planned, it should load the structure and function appropriately, and the stress in the tissue should be at a level that provides positive effects and contributes to progress [6]. When hyolaryngeal elevation is desired, the characteristics of the suprahyoid muscles and physiology of swallowing are well known. Considering the architectural features of the suprahyoid muscles, eccentric exercise offers a new perspective because some suprahyoid muscles have long sarcomere lengths [7–9]. The appropriate exercise method to be used in strengthening muscles with a long sarcomere is eccentric exercise [10,11]. It is very complicated to apply eccentric exercise since the suprahyoid muscles do not settle around a joint and the insertion (hyoid bone) does not remain stable during function.

By understanding the potential for hyoid excursion resulting from the structural properties of these muscles, therapists can target the submental muscles with exercises designed to promote their capacity. To the best of our knowledge, there are two eccentric exercise models described in the dysphagia literature: the Modified Jaw Opening Exercise (MJOE) and the Submandibular Push Exercise (SMPE) [12,13]. Both exercises were performed with the mouth closed and without any mandibular or neck movements. Both studies reported that eccentric contractions occur during the static position by trying to open the mouth against resistance coming from under the chin or by pushing the submandibular region down this resistance. Eccentric contraction is part of isotonic contraction. Isotonic contraction in the static position can only occur at minimal angles, which is very similar to isometric contractions. Although eccentric contractions are the exercise form with the highest potential for muscle strength production [7], isometric exercises provide muscle strength only at the angle they are performed [14]. In addition, dynamic full range of motion (ROM) exercises have more positive effects on muscle cross-sectional area, fascicle length, and isometric strength at all angles [15]. Among the dynamic ROM exercises, Shaker and Chin-Tuck Against Resistance (CTAR) exercises are the most common exercises in the literature for hyolaryngeal elevation. These exercises aim to increase hyolaryngeal elevation with head flexion, facilitate relaxation of the cricopharyngeal muscle [16], and increase suprahyoid muscle activation [17]. A common feature of these exercises is that they are both performed with isotonic and isometric contractions. In the literature on dysphagia, eccentric exercises with dynamic ROM have been lacking.

Eccentric Chin Closure (ECC) exercise is a previously proposed and designed exercise that follows the principles of eccentric contraction [7]. It is a dynamic ROM eccentric exercise starting from maximum mouth opening and closing the chin against the resistance given under the chin. Although the ECC exercise has been previously introduced theoretically, a clinical study has not yet been conducted. is study aim is to investigate the effects of ECC exercise on submental muscle activation, muscle strength, dysphagia limit, perceived exertion and pain compared with Shaker and CTAR exercises in healthy volunteers.

## Materials and methods

### Study design

In this prospective, randomized parallel group study, the effects of ECC exercise in healthy volunteers were compared with those of Shaker and CTAR exercises.

The study was conducted according to the Declaration of Helsinki guidelines, and ethical approval was obtained from the Hacettepe University Clinical Research Ethics Committee (Approval Number: KA-21002/2023/01-01). Written informed consent was obtained from all the volunteers. An experienced expert (first author) with eight years of experience conducted all interventions and data collection at Hacettepe University, Faculty of Physical Therapy and Rehabilitation, Swallowing Disorders Unit and Hacettepe University Hospitals, Department of Neurology between May 16, 2023 and September 23, 2023. This study was registered in a clinical trial database (registration number: NCT05240599).

### Participants

Although ECC exercise has been theoretically introduced before, this study aimed to investigate the effects of ECC exercise on submental muscle activation, muscle strength, dysphagia limit, perceived exertion and pain compared with Shaker and CTAR exercises. Therefore, a group of healthy young adults was preferred to determine the efficacy of the ECC exercise before its application in patients.

**Fig 1:**
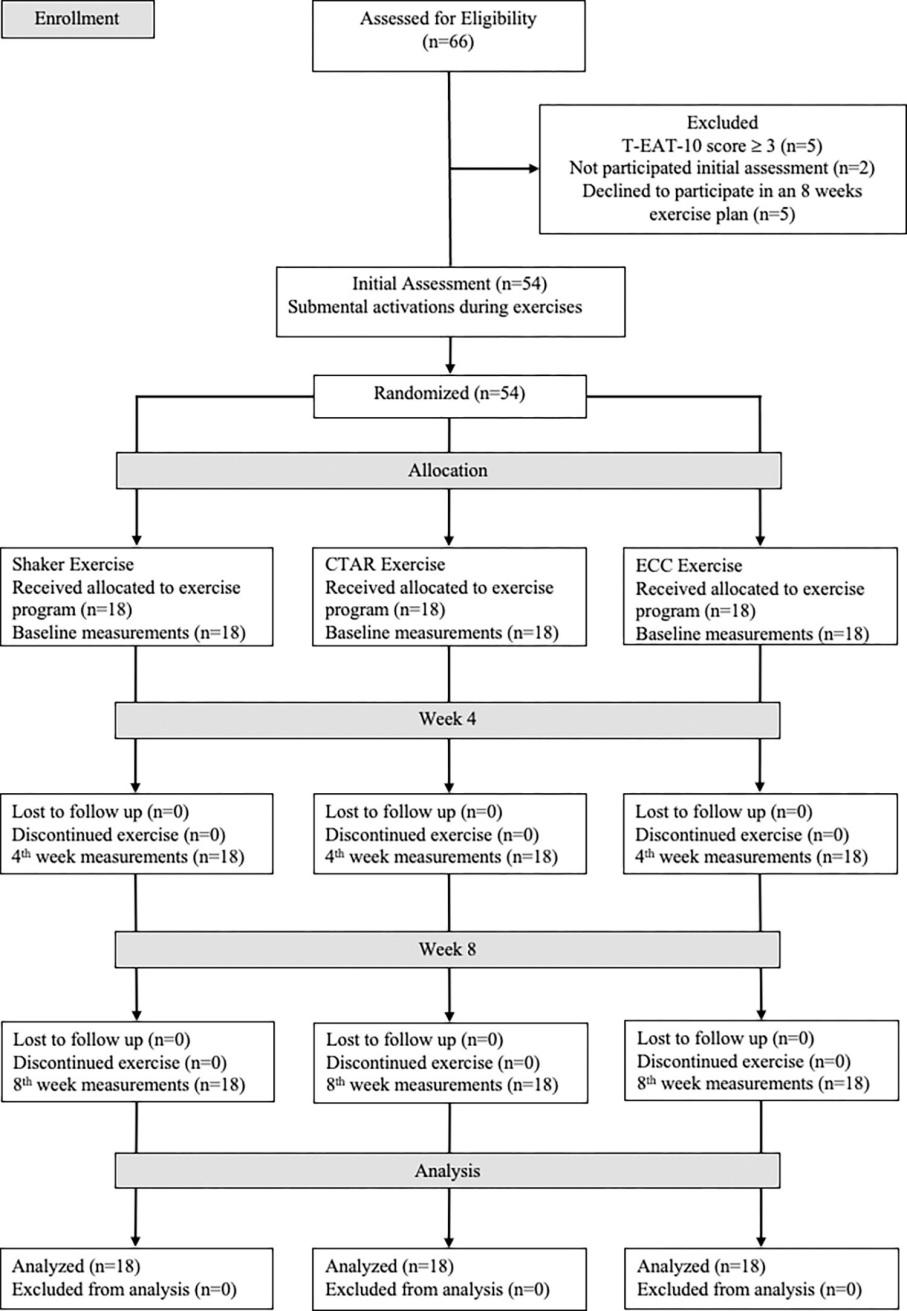
CONSORT flow diagram.

Sixty-six healthy volunteers aged between 18–35 years were enrolled in this study. Volunteers completed the Turkish version of the Eating Assessment Tool (T-EAT-10) questionnaire, and those who scored more than 3 points were excluded from the study (n = 5). The T-EAT-10 questionnaire assesses dysphagia symptom severity, and scores below three points indicate normal swallowing [18]. Other exclusion criteria were having any pain, pathology, radiotherapy, surgery, or other pathologies in the head and neck region, temporomandibular joint problems, or neurological or systemic disease (n = 0). Volunteers who also did not participate in the assessments (n = 2), declined to participate 8 weeks of exercise plan (n = 5) and missed five or more days of weekly exercise follow-up charts (n = 0) were excluded from the study. 54 volunteers were included in the initial assessment of the study. Following the initial assessment, 54 volunteers aged between 19–28 were randomly allocated into three groups, a Shaker exercise group (n = 18), CTAR exercise group (n = 18) and ECC exercise group (n = 18). Randomization stratified by gender, and allocation ratio was 1:1:1. Randomization was performed by a researcher who had no contact with volunteers (Fig 1). After randomization, all volunteers started the exercise programs at the same time and completed the study in originally assigned groups without loss and exlusion. The study ended with the completion of the 8-week exercise program. All volunteers participated in the assessments at baseline and measurement times, and there were no missing data.

### Exercises

In this study, volunteers were assigned to ECC exercise, Shaker, and CTAR groups, and the effects of the exercises on submental muscle activation, muscle strength, dysphagia limit, perceived exertion, and pain were investigated.

#### Eccentric Chin Closure (ECC) exercise

The ECC exercise is designed to produce an eccentric contraction against resistance from the shortest to the longest position of the suprahyoid muscles. It is an exercise that uses eccentric manual resistance adjusted to 60–80% of the 1 repetition maximum (1-RM) given under the chin starting from the maximum mouth opening through closing.

**Amount of resistance:** First, maximum suprahyoid muscle strength (1-RM) was measured with a digital dynamometer (Jtech Medical Industries Commander Muscle Testing 7633s). Then 60–80% of the 1-RM was calculated. Volunteers manually applied the calculated amount of loading to their thumbs during exercise.

**Orientation to resistance:** Volunteers were instructed to exert this calculated amount of force by pressing the same dynamometer with their preferred thumb while observing the dynamometer’s screen. The volunteers were given the time to practice. Orientation to the amount of resistance was considered to be achieved when volunteers successfully performed the required force within the desired range three times in a row, without looking at the screen. Notably, all volunteers were able to achieve an orientation towards the amount of resistance.

**Fig 2:**
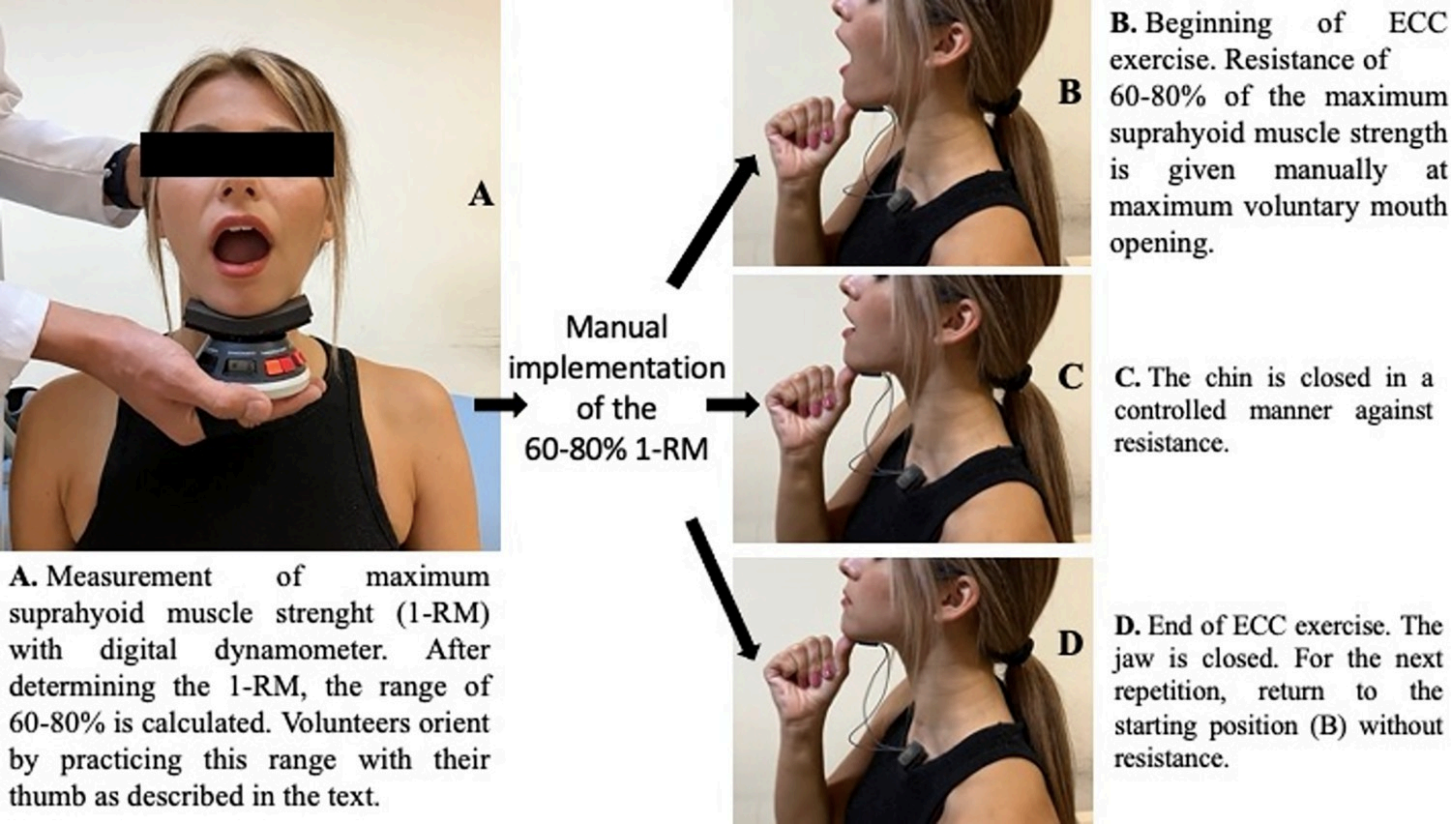
ECC exercise.

**Implementation:** Volunteers were asked to sit upright on a backsupported chair. In addition to minimal hyoid movement in the posterior-inferior direction during maximum mouth opening, the distance between the hyoid and tip of the mandible was significantly shortened. [19]. Therefore, maximum mouth opening was chosen as the starting position for the eccentric exercise. The volunteers were asked to open their mouths as much as possible. The volunteer was then asked to place the thumb on the tip of the mandible and apply the resistance that had previously worked on the dynamometer in the direction of chin closure. Finally, the volunteer was asked to close the chin against upward resistance in a controlled manner, starting from maximum mouth opening. They were also instructed that the resistance should be met with muscle force and should not help with chin closure (Fig 2).

#### Shaker exercise

The volunteers were instructed to lie on their backs, with their knees straight. In the isometric component, the volunteers were asked to lift their heads without lifting their shoulders and look at their toes for 60 s. In the isotonic component, the volunteers were directed to raise their heads without lifting their shoulders, look at the tip of their feet, and return to the starting position without pausing [16].

#### Chin-Tuck Against Resistance (CTAR) exercise

Volunteers were instructed to place an inflatable ball with a diameter of 12 cm between their chin and sternum, while sitting upright on a back-supported chair. In the isometric component, volunteers were asked to tuck the chin, compress the ball with maximum force between their chin and sternum, and hold it for 60 seconds. For the isotonic component, the volunteers were instructed to squeeze the ball between their chin and sternum with the maximum force they could exert [17].

### Dose

Shaker, CTAR, and ECC exercises were designed in accordance with the needs of the target muscle group (suprahyoid muscles) to increase muscle strength and muscle activation. In the literature on dysphagia, the optimal dose parameters for exercise-based applications remain unclear. Understanding of the design and delivery of exercise-based training regimens is required [20,21]. In this study, exercise training was planned by considering the number of repetitions, frequency, duration, and intensity as the dose parameters.

**Repetitions**: The exercises were structured as 10 repetitions for CTAR and ECC. The shaker exercise was performed in 30 consecutive repetitions in accordance with the protocol. The Shaker and CTAR exercises also have isometric components. The isometric components of the CTAR and Shaker exercises were set at 60 seconds in this study.

**Intensity:** It was not possible to measure the amount of loading during the Shaker and CTAR exercises. In the Shaker exercise, volunteers performed the exercise against the weight of their head, whereas the CTAR exercise used a standard ball with a diameter of 12 cm. The 1 repetition maximum (1-RM) method was used in the ECC exercise. 60–80% of 1 maximum repetition was determined as the amount of loading. Although there are various applications for each muscle and function in the literature, loads over 80% of 1-RT increase maximum strength, loads between 60–65% and 80% increase strength and endurance, and loads below 65% increase endurance [22]. In the dysphagia literature, the 1-RM method has been used for expiratory muscle strength training and lingual strength training [23–25]. In lingual strength training, 60% of 1-RM in the first week and 80% in the following weeks were set as the amount of resistance [24]. Since no training device was used where the desired percentage could be precisely adjusted in this study, in the ECC exercise, the amount of resistance was adjusted to be 60–80% of the maximum suprahyoid muscle strength. Resistance amount was measured and updated weekly.

**Frequency:** In accordance with the CTAR and Shaker exercise protocols, all exercises were planned in 3 sets. Taking the Shaker exercise protocol as a reference, the rest period between sets was 60 s.

**Duration:** The exercise science literature recommends 8–12 week programs for resistance training [26] and exercise training in this study was applied for 8 weeks.

**Implementation:** Fifty-four volunteers were trained to perform the exercises for 20 min before the initial assessments and performed all three exercises. After the initial measurements were completed, fifty-four volunteers were randomly assigned to their own exercise group and practiced the exercises three times a day for eight weeks as a home exercise program. Exercise adherence of the volunteers was monitored and recorded on a weekly follow-up chart.

### Data collection

#### Electromyographic measurements

Dual-channel DELSYS Trigno Duo sensors integrated into the software DELSYS Trigno Lite System were used for surface electromyography (sEMG) measurements. The skin surface was cleaned using alcohol swabs. Wireless DELSYS Trigno Duo sensors were symmetrically attached to the submental area between the hyoid bone and mandible, with centers separated by 20 mm [27]. A ground electrode was attached to the skin of the left clavicle. Submental muscle activation was recorded in volts (V) (Fig 2). The sEMG signals were recorded at 1500 Hz. The data were analyzed using Delsys EMG Works. A bandpass filter was applied to the sEMG signals (high filter pass, 10 Hz; low filter pass, 500 Hz), with a notch filter of 60 Hz [28].

*Submental muscle activity during exercises*. As an initial assessment in this study, submental sEMG activity was measured once during the exercises before the 8-week program. 54 volunteers performed all exercises using an electromyography device. Since the ECC exercise did not include an isometric component, sEMG data were recorded during eccentric exercise for ECC and isotonic exercise for Shaker and CTAR exercises. These data were recorded to examine the potential of these three exercises to produce muscle activation in untrained volunteers. The exercises were performed in a mixed order to prevent the order of exercise froms causing BIAS and to develop adaptation. Each exercise was performed according to its own protocol and a 5-minute rest break was provided between exercises.

**Fig 3:**
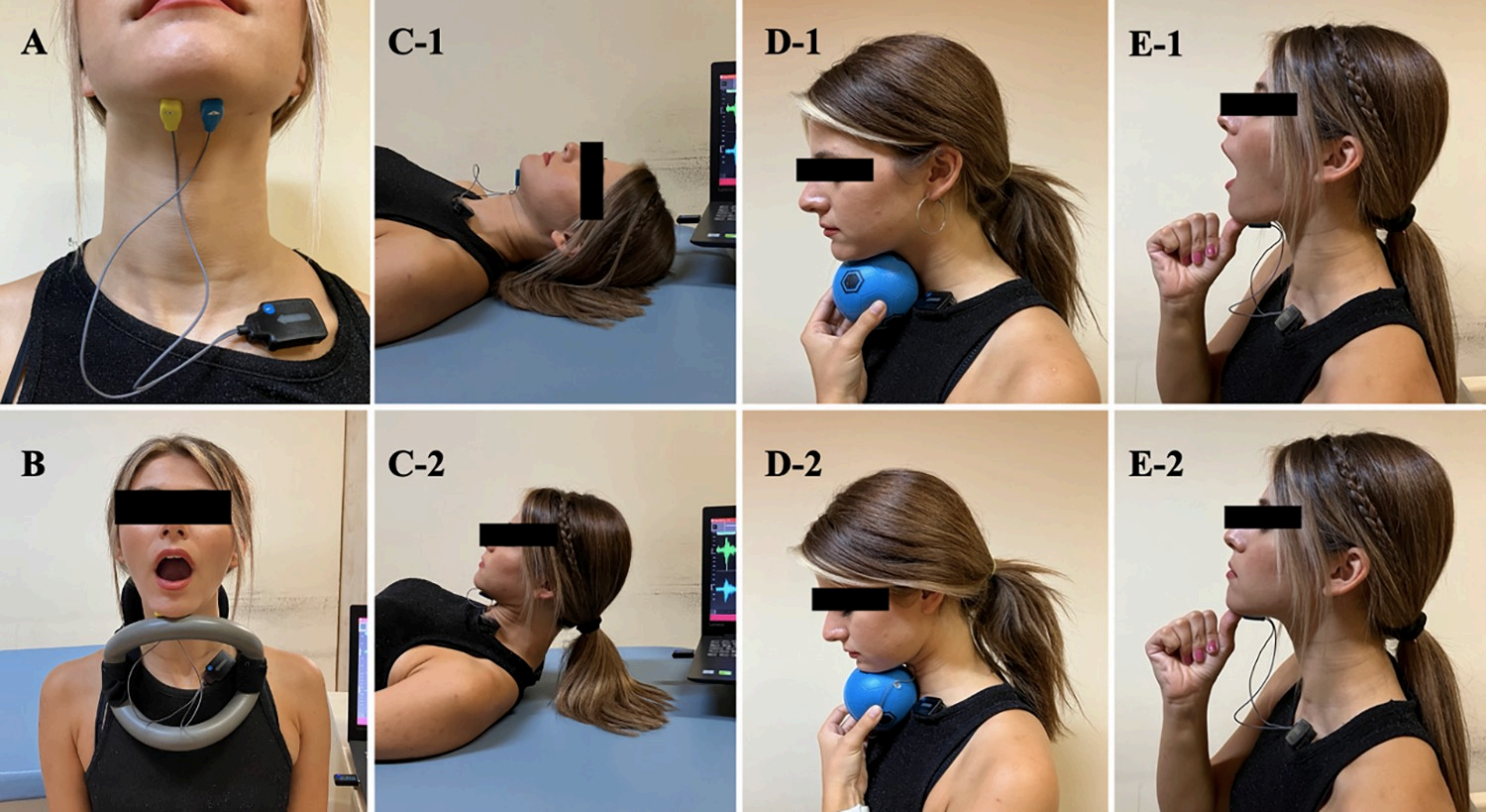
sEMG data collection process. **A.** sEMG placement, **B.** MVC measurement, **C1-2** initial and final positions of Shaker exercise, **D1-2** initial and final positions of chin-tuck against resistance (CTAR) exercise, **E1-2** initial and final positions of eccentric chin closure (ECC) exercise.

*Maximum voluntary isometric contraction (MVC)*. sEMG measurements were performed in all volunteers during MVC as described by Kılınç et al. [29]. The volunteers were instructed to sit upright on a back-supported chair. To measure MVC, a semi-rigid neck orthosis that allows only mouth opening was used [29]. Volunteers were asked to press their chin down for 10 s against the cervical neck orthosis, repeating this action five times with a 60-second rest interval between each contraction (Fig 3). The root mean square of the raw amplitude was calculated as the index of muscle activity, and the highest value was recorded as the MVC. This measurement was repeated at the baseline, 4th, and 8th weeks. MVC was used to normalize the muscle activation recorded during the activity and to determine the change in MVC capacity before and after exercise.

*Normalization procedure*. sEMG data need to be normalized, as it is insufficient to compare the muscle activation levels achieved during exercise among volunteers [30]. When normalization is not performed, activations of the same individual for different activities can be compared. Normalization is essential for statistically comparing sEMG data obtained from different individuals [30]. Although there are various normalization methods, the MVC method is the most commonly used [30]. In this method, the activation during MVC represents the maximum electrical muscle activation produced during an activity. In this study, muscle activation obtained during exercise was compared to MVC. The normalization procedure was performed using the EMG Works Analysis software as follows: Maximum Electrical Activity During Exercise/Maximum Electrical Activity During MVC. The results were saved as the MVC%.

#### Muscle strength

Volunteers were asked to sit upright on a chair. The head of the digital dynamometer (Jtech Medical Industries Commander Muscle Testing 7633s) was placed under the chin of the volunteer, who was then asked to open their mouth vigorously for 10 seconds against the dynamometer. The dynamometer was held steady throughout the measurement period. This measurement was repeated three times, with a 60-second resting period in between, and the maximum values were recorded in Pound-Force (lbs). (Fig 2A). Muscle strength measurements were repeated at baseline, 4th and 8th week. Muscle strength data were used to determine the amount of resistance and change in muscle strength (1-RM) before and after the exercise.

#### Dysphagia limit

The volunteers received increasing volumes of water (1, 3, 5, 10, 15, 20, 25, and 30 ml) via glass. Electrical signals were monitored during water consumption and swallowing was marked by a burst of sEMG activity. The volume of water that the volunteers could not drink in a single swallow was identified as the dysphagia limit [29]. Normal piecemeal deglutition in healthy individuals and patients without dysphagia has been reported to be 20 ml. [31]. The test was terminated if the volunteer exhibited signs of piecemeal swallowing or subglottic aspiration.

#### Perceived exertion and pain

Volunteers performed their own exercises under the supervision of the researcher at baseline, week four, and week eight. Volunteers were assessed for perceived exertion and pain immediately after the exercise. The Borg Rating of Perceived Exertion (RPE) scale was used for exertion, ranging from 6 to 20 [32]. A score of 6 indicates no fatigue or strain, whereas 20 indicates the highest level of fatigue. Pain caused by exercise was evaluated using a visual analog scale [33]. Volunteers marked the severity of pain on a 10 cm-long line, with the length of the scale representing the score. A score of 0 indicated no pain and 10 indicated very severe pain.

### Sample size estimation

Sample size was calculated using G-Power software version 3.1.2. According to 5% type 1 error, two-way hypothesis design and CTAR values obtained in the literature [34], the effect size was determined as 0.78. According to these values and the primary hypothesis of current study, “There is no difference between CTAR, Shaker and ECC exercises in terms of submental muscle activation, muscle strength, dysphagia limit, perceived exertion and pain level after 8 weeks of exercise.”, it was calculated that 16 volunteers were needed in each group in order to obtain at least 80% power. Considering the 10% non-response rate, a higher sample size targeted (n = 18 per group) and the study was completed with the participation of 18 volunteers in each group.

### Statistical analysis

Data were analyzed using the statistical package program IBM SPSS Statistics Standard Concurrent User V 26 (IBM Corp., Armonk, New York, USA). Descriptive statistics are given as number (*n*), percentage (%), mean (*X*), standard deviation (*SD*), median (*M*), minimum (*min*) and maximum (*max*) values. The normal distribution of the data of quantitative variables was determined by examining the skewness and kurtosis values. At the decision stage, if the absolute Skewness value is below ± 2.0 and the Kurtosis value is below 7.0, it is decided that the data are normally distributed [35]. The quantitative descriptive characteristics and variables were found to be normally distributed and parametric tests were used.

One-way ANOVA (analysis of variance) was used to compare quantitative descriptive characteristics and measurements of volunteers among groups, and chi-square tests (Pearson chi-square/Fisher exact test) were used to compare categorical descriptive characteristics among groups.

Measurements at baseline, 4th week, and 8th week were compared among the groups, according to group, time, and group*time interaction and change in the mean scores compared within groups using Mixed Design ANOVA. In this study, the effect size for the 8-week changes in MVC values across the groups was calculated to be η^2^ = 0.220 at a 5% significance level for a sample of 54 volunteers. The statistical power for this effect size was determined to be 99.2%. Similarly, for the same sample size and significance level, the effect size for the 8-week changes in the MS was found to be η^2^ = 0.210, with the corresponding power calculated at 99.4%. Post hoc analyses were performed using the Bonferroni-Dunn test to reveal statistical differences in the groups. In addition, post hoc analyses were performed using the Bonferroni-Dunn test to reveal statistically significant differences at Baseline, 4th week and 8th week. Statistical significance was set at *p<*0.05.

The intra- and inter-rater reliabilities of the electromyographic measurements of maximum voluntary isometric contraction on 20% of the data of each exercise group were analyzed using intra-class correlation coefficients (ICC). For the results values below 0.5 were considered as poor reliability, values between 0.5 and 0.75 as moderate reliability, values between 0.75 and 0.9 as good reliability, and values above 0.9 as excellent reliability [36].

## Results

A total of 54 volunteers who met the inclusion criteria, 32 (59.3%) women and 22 (40.7%) men, aged–19–28 (mean = 22.00 ± 1.91) years, were included in the analysis. After initial assessment, 54 people were randomized into 3 groups. Each group consisted of healthy young adults who scored 3 points or less on the T-EAT-10, had no pain, pathology, radiotherapy, surgery, and other pathologies in the head and neck region, no temporomandibular joint problems, and no neurologic or systemic disease. Distribution of the descriptive characteristics of the volunteers according to groups are showed in Table 1. There were 54 participants in the study; 18 in the Shaker group, 18 in the CTAR group, and 18 in the ECC group. There were 7 (38.9%) male participants in the Shaker group, 7 (38.9%) in the CTAR group and 8 (44.4%) in the ECC group. The median age of the volunteers was 22 years in the Shaker group, 21 years in the CTAR group and 22 years in the ECC group. Mean height was 170.56 ± 10.95 cm in the Shaker group, 21.61 ± 1.88 cm in the CTAR group and 170.00 ± 9.54 cm in the ECC group. Mean weight was 63.56 ± 14.07 kg in the shaker group, 63.22 ± 9.33 kg in the CTAR group and 63.61 ± 10.91 kg in the ECC group. Mean BMI was 21.64 ± 2.93 kg/m2 in the Shaker group, 22.10 ± 2.45 kg/m2 in the CTAR group and 21.92 ± 2.58 kg/m2 in the ECC group. The descriptive characteristics of the volunteers in the study groups showed a similar distribution (*p>0*.05).

**Table 1:** Comparison of descriptive characteristics of participants according to groups (*n =* 54).

	Group		
	Shaker	CTAR	ECC
	*n =* 18	*n =* 18	*n =* 18
**Gender**, *n* (*%*)			
Male	7 (38.9%)	7 (38.9%)	8 (44.4%)
Female	11 (61.1%)	11 (61.1%)	10 (55.6%)
**Age**, (year)			
*X* ± *SD*	21.78 ± 1.59	21.61 ± 1.88	22.39 ± 2.23
*M* (*min*-*max*)	22 (19–26)	21 (19–26)	22 (20–28)
**Height**, (*cm*)			
*X* ± *SD*	170.56 ± 10.95	169.00 ± 9.57	170.00 ± 9.54
*M* (*min*-*max*)	168.5 (155–195)	167 (157–185)	171.5 (155–184)
**Weight**, (*kg*)			
*X* ± *SD*	63.56 ± 14.07	63.22 ± 9.33	63.61 ± 10.91
*M* (*min*-*max*)	62.5 (41–101)	59.5 (50–78)	60 (50–90)
**Body Mass Index**, (*kg/m*^*2*^)			
*X* ± *SD*	21.64 ± 2.93	22.10 ± 2.45	21.92 ± 2.58
*M* (*min*-*max*)	21.9 (17–26.6)	22 (19–28.5)	21.4 (18.9–27.2)

Descriptive statistics given as: *Mean* (*X*), *standard deviation* (*SD*), *median* (*M*), *minimum* (*min*), *maximum* (*max*), *count* (*n*), *percent* (*%*).

The mean exercise adherence percentage was 81.05 ± 5.87 in the Shaker group, 80.03 ± 7.65 in the CTAR group and 80.99 ± 7.87 in the ECC group. Mean exercise adherence was statistically similar among the exercise groups (*p>0*.05).

The intra- and inter-rater reliabilities of sEMG activations of MVC at baseline at 4^th^ and 8^th^ weeks are presented in Table 2.

**Table 2:** Intra-rater and inter-rater reliability of sEMG activations of MVC.

	Baseline	4^th^ Week	8^th^ Week
**Intra-rater Reliability**	**ICC (%95 CI)**	**ICC (%95 CI)**	**ICC (%95 CI)**
**Shaker**	0.97 (0.69–0.99)	0.88 (0.80–0.99)	0.99 (0.98–1)
**CTAR**	0.91 (-0.43–0.99)	0.97 (0.58–0.99)	0.99 (0.99–1)
**ECC**	0.99 (0.99–1)	0.99 (0.90–1)	0.99 (0.92–1)
**Inter-rater Reliability**	**ICC (%95 CI)**	**ICC (%95 CI)**	**ICC (%95 CI)**
**Shaker**	0.95 (0.24–0.99)	0.94 (0.39–0.99)	0.98 (0.65–0.99)
**CTAR**	0.78 (-0.59–0.97)	0.81 (-0.60–0.99)	0.88 (-0.33–0.99)
**ECC**	0.82 (-0.64–0.99)	0.85 (-0.21–0.96)	0.89-0.69–0.99)

ICC: Intraclass correlation coefficient; CI: Confidence interval.

### Initial assessment: Submental muscle activation during exercises

Table 3 shows that the mean submental muscle activation recorded during the exercises was 44.37 ± 21.72 in the shaker group, 61.60 ± 22.05 in the CTAR group and 62.19 ± 24.34 in the ECC group. The mean submental muscle activation recorded during exercise in the shaker group was statistically lower than the CTAR and ECC groups (*p<0*.05).

**Table 3:** Comparison of normalized submental muscle activation (%MVC) during exercises (*n =* 54).

	Shaker	CTAR	ECC	Test (*p*)
**%MVC**				***F =* 22.027 *p<*0.001**
*X* ± *SD*	44.37 ± 21.72 ^*b*^	61.60 ± 22.05 ^*a*^	62.19 ± 24.34 ^*a*^	
*M* (*min*-*max*)	36.7 (15.6–94.2)	64.4 (18.0–98.1)	56.9 (22.4–99.4)	

Repeated Measures ANOVA (F*); Descriptive statistics given as: *Mean* (*X*), *standard deviation* (*SD*), *median* (*M*), *minimum* (*min*), *maximum* (*max*), *count* (*n*), *percent* (*%*). *a>b*: Differences between different letters in the same row or column are significant (*p<*0,05).

### Follow-up measurements

**Fig 4:**
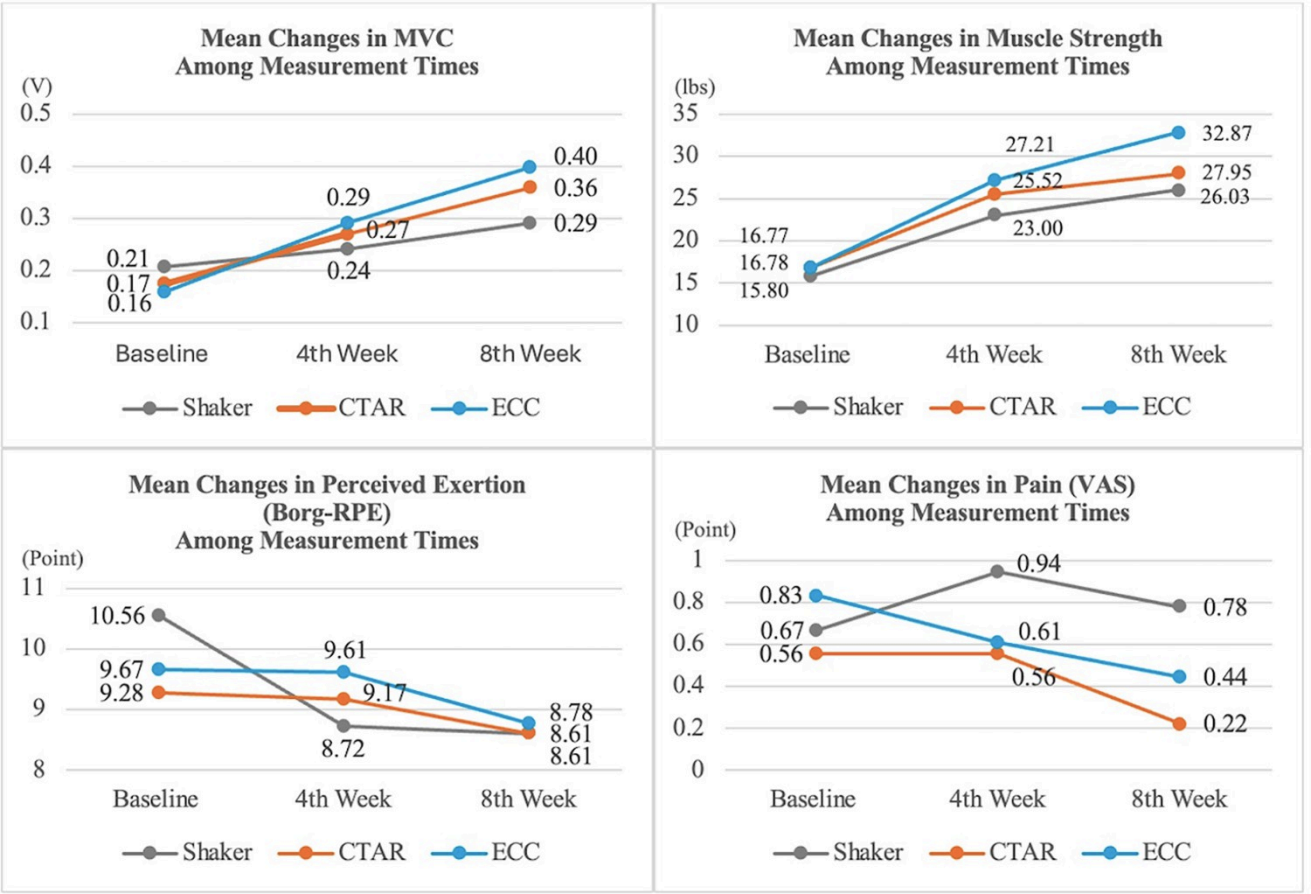
Mean changes on MVC, muscle strength, perceived exertion and pain.

Table 4 presents a comparison of MVC, muscle strength, perceived exertion, and pain measurements among the three groups at follow-up times. The mean changes in MVC, muscle strength, perceived exertion, and pain are illustrated in Fig 4. All volunteers successfully completed the dysphagia limit protocol at baseline, 4th, and 8th weeks, with no instances of piecemeal deglutition below 30 ml observed. Consequently, dysphagia limit was not included in the outcome measures.

**Table 4:** Comparison of MVC, muscle strength, perceived exertion and pain measurements by groups at follow-up times (*n =* 54).

	Group			Test Statistics ^†^	Model Significance		
	Shaker	CTAR	ECC		Group Effect	Time Effect	Group X Time Effect
	*n =* 18	*n =* 18	*n =* 18				
**MVC**					*F =* 0.384 *p =* 0.683 *η*^*2*^ *=* 0.015	***F =* 95.932 *p<*0.001 *η***^***2***^ ***=* 0.653**	***F =* 7.203** ***p<*0.001 *η***^***2***^ ***=* 0.220** **Power = 0.992**
*Baseline*	0.21 ± 0.15 ^*bc*^	0.17 ± 0.08 ^*c*^	0.16 ± 0.13 ^*c*^	*F =* 0.725 *p =* 0.489 *η*^*2*^ *=* 0.028			
*4th Week*	0.24 ± 0.17 ^*b*^	0.27 ± 0.09 ^*b*^	0.29 ± 0.11 ^*b*^	*F =* 0.691 *p =* 0.506 *η*^*2*^ *=* 0.026			
*8th Week*	0.29 ± 0.19 ^*a*^	0.36 ± 0.10 ^*a*^	0.40 ± 0.14 ^*a*^	*F =* 2.325 *p =* 0.108 *η*^*2*^ *=* 0.084			
Test Statistics ^ϕ^	***F =* 5.398 *p =* 0.008 *η***^***2***^ ***=* 0.178**	***F =* 25.601 *p<*0.001 *η***^***2***^ ***=* 0.506**	***F =* 42.488 *p<*0.001 *η***^***2***^ ***=* 0.630**				
**MS**					*F =* 2.016 *p =* 0.144 *η*^*2*^ *=* 0.073	***F =* 324.243 *p<*0.001 *η***^***2***^ ***=* 0.864**	***F =* 6.786** ***p<*0.001 *η***^***2***^ ***=* 0.210** **Power = 0.994**
*Baseline*	15.80 ± 4.27 ^*c*^	16.78 ± 5.84 ^*d*^	16.77 ± 7.75 ^*d*^	*F =* 0.151 *p =* 0.861 *η*^*2*^ *=* 0.006			
*4th Week*	23.00 ± 5.99 ^*c*^	25.52 ± 6.89 ^*c*^	27.21 ± 7.15 ^*c*^	*F =* 1.801 *p =* 0.176 *η*^*2*^ *=* 0.066			
*8th Week*	26.03 ± 5.86 ^*b*^	27.95 ± 6.33 ^*b*^	32.87 ± 6.55 ^*a*^	***F =* 5.727 *p =* 0.006 *η***^***2***^ ***=* 0.183**			
Test Statistics ^ϕ^	***F =* 80.736 *p<*0.001 *η***^***2***^ ***=* 0.764**	***F =* 79.752 *p<*0.001 *η***^***2***^ ***=* 0.761**	***F =* 230.186 *p<*0.001 *η***^***2***^ ***=* 0.902**				
**Borg-RPE**					*F =* 0.115 *p =* 0.892 *η*^*2*^ *=* 0.004	***F =* 3.974 *p =* 0.022 *η***^***2***^ ***=* 0.072**	*F =* 1.044 *p =* 0.388 *η*^*2*^ *=* 0.039 Power = 0.319
*Baseline*	10.56 ± 2.81 ^*a*^	9.28 ± 3.12 ^*ab*^	9.67 ± 3.38 ^*ab*^	*F =* 0.797 *p =* 0.456 *η*^*2*^ *=* 0.03			
*4th Week*	8.72 ± 2.82 ^*b*^	9.17 ± 3.11 ^*ab*^	9.61 ± 2.06 ^*ab*^	*F =* 0.487 *p =* 0.617 *η*^*2*^ *=* 0.019			
*8th Week*	8.61 ± 3.11 ^*b*^	8.61 ± 2.45 ^*ab*^	8.78 ± 2.51 ^*ab*^	*F =* 0.023 *p =* 0.978 *η*^*2*^ *=* 0.001			
Test Statistics ^ϕ^	***F =* 3.712 *p =* 0.031 *η***^***2***^ ***=* 0.129**	*F =* 0.544 *p =* 0.584 *η*^*2*^ *=* 0.021	*F =* 1.099 *p =* 0.341 *η*^*2*^ *=* 0.042				
**VAS**					*F =* 0.498 *p =* 0.611 *η*^*2*^ *=* 0.019	*F =* 0.645 *p =* 0.527 *η*^*2*^ *=* 0.012	*F =* 0.346 *p =* 0.846 *η*^*2*^ *=* 0.013 Power = 0.126
*Baseline*	0.67 ± 1.64	0.56 ± 1.38	0.83 ± 1.58	*F =* 0.148 *p =* 0.862 *η*^*2*^ *=* 0.006			
*4th Week*	0.94 ± 1.70	0.56 ± 1.50	0.61 ± 1.38	*F =* 0.339 *p =* 0.714 *η*^*2*^ *=* 0.013			
*8th Week*	0.78 ± 1.48	0.22 ± 0.73	0.44 ± 0.92	*F =* 1.183 *p =* 0.315 *η*^*2*^ *=* 0.044			
Test Statistics ^ϕ^	*F =* 0.255 *p =* 0.776 *η*^*2*^ *=* 0.01	*F =* 0.937 *p =* 0.399 *η*^*2*^ *=* 0.036	*F =* 0.579 *p =* 0.564 *η*^*2*^ *=* 0.023				

Mixed ANOVA (*F*), Effect Size (***η***^***2***^), ^ϕ^ Intra-group comparison, ^**†**^ Inter-group comparison, Descriptive statistics given as: *Mean* (*X*), *standard deviation* (*SD*). Bolded sections are statistically significant (*p<*0.05). *a>b>c>d*: Differences between different letters in the same row or column are significant (*p<*0.05).

The mean MVC values did not reveal a statistically significant difference among the groups (*p>*0.05). However, in the Shaker group, the mean MVC at week 8 was significantly higher than at baseline and 4th week (*p<*0.05). In the CTAR and ECC groups, the mean MVC increased significantly at all measurement times (*p<*0.05). The 8 weeks of exercise program was effective in enhancing MVC activations across all groups. Considering the groupXtime effect, MVC increased significantly more in the CTAR and ECC groups than in the Shaker group (F = 7.203, *p<*0.001).

The mean muscle strength values at the baseline and 4th week measurements did not show statistically significant differences among the groups (*p>*0.05). At the 8th week, the mean muscle strength in the ECC group was significantly higher compared to the Shaker and CTAR groups (*p<*0.05). In the Shaker group, the mean muscle strength at the 8th week was significantly greater than at baseline and 4th week (*p<*0.05). In both the CTAR and ECC groups, mean muscle strength increased significantly at all measurement times (*p<*0.05). Consequently, the 8 weeks of exercise program was effective in enhancing muscle strength across all groups. When considering groupXtime effects, muscle strength increased more in the ECC group than in the Shaker and CTAR groups (F = 6.786, *p<*0.001).

The mean Borg-RPE, which indicates perceived exertion levels, did not show a significant difference among the groups (*p>*0.05). In the Shaker group, the mean Borg-RPE at 4th and 8th week were significantly lower than that at baseline (*p<*0.05). No significant intra-group differences were observed between the CTAR and ECC groups (*p>*0.05). Borg-RPE values showed similar changes across all three groups (F = 1.044, p = 0.388).

The mean VAS values indicating the pain levels of the volunteers at the measurement times did not show statistically significant difference between intra-group and inter-groups (*p>0*.05). The changes in the VAS values in the Shaker, CTAR, and ECC groups were not statistically significant (*F = 0*.346, *p = 0*.846).

## Discussion

In the current study, the effectiveness of ECC exercise on submental muscle activation, muscle strength, perceived exertion, and pain levels was compared to CTAR and Shaker exercises. The initial assessment results revealed that the Shaker exercise showed lower submental muscle activation than the ECC and CTAR exercises in untrained volunteers. After the exercise program, it was found that exercise training provided similar improvements at the 4th week. The results of the 8-week exercise program demonstrated that all three exercises had positive effects on muscle activation, muscle strength, perceived exertion, and pain. As a result, while all exercise groups showed improvements in MVC activation and muscle strength after 8 weeks, the CTAR and ECC exercises were more effective than the Shaker exercise in increasing MVC. Additionally, the ECC exercise demonstrated superior efficacy in enhancing muscle strength compared to both the Shaker and CTAR exercises at the 8th week, with similar levels of perceived exertion and pain observed across all groups.

Skeletal muscle meets its energy needs during activity in two different systems: anaerobic and aerobic. During muscle contraction, the energy needs are first met by the creatine phosphate system of the anaerobic system. This system uses energy stored in the muscle for 4–10 seconds. From the 10th second to 2 min, the glycolytic system, which is also anaerobic, is activated. After 2 min, the aerobic system is activated and oxygen is required to maintain muscle function [37]. According to the literature on dysphagia, it is thought that the anaerobic system should be trained to generate the force required for swallowing. It has also been stated that the aerobic system should be supported to provide the endurance required for repeated swallowing during a meal [37]. The muscles involved in swallowing predominantly contain type II fibers. Type-IIx fibers have the highest power-generating capacity, whereas Type-IIa fibers are rich in glycolytic enzymes and fuel for anaerobic system. These fibers are also called fast-oxidative fibers, and can use both aerobic and anaerobic metabolism owing to their rapid adaptability [20]. They are also the most affected fibers in conditions, such as sarcopenia. Therefore, in this study, a regimen of exercises was structured to support Type II muscle fiber properties and metabolic needs. The goal of this study was to increase muscle activation and muscle strength by promoting anaerobic metabolism. After the 8-week exercise program, it was concluded that the 3 exercises achieved these goals at different rates.

In this study, ECC and CTAR exercises had similar isotonic submental activations during exercise and MVC after 8-week exercise training, while the Shaker exercise showed lower activation than both ECC and CTAR exercises. This finding aligns with previous studies comparing CTAR and Shaker exercises, which have shown that the CTAR exercise elicits greater submental sEMG activation and MVC than the Shaker exercise [17,38]. In this study, ECC and CTAR exercises showed similar activation levels both at the initial assessment and after 8 weeks of exercise. Similar to our study, Kılınç et al. showed that an 8-week CTAR and chin tuck exercise with Theraband exercises were superior to Shaker in terms of MVC [29]. The similarity in activation levels between ECC and CTAR exercises may be attributed to both exercises targeting suprahyoid muscle activation. It is known that neck flexors, such as the sternocleidomastoid muscle, are also involved in muscle activation during Shaker exercise. Studies have shown that the Shaker exercise produces more sternocleidomastoid activation and less submental muscle activation than CTAR exercise [17,38]. Although the current study did not include sEMG measurements of other muscles, the ECC exercise does not involve neck flexion and, as a full ROM exercise, may offer a focused approach on the suprahyoid muscles similar to CTAR. From another perspective, eccentric loading is known to provide more motor unit firing [39] and Chang MC et al. reported that SMPE, which was also introduced as an eccentric exercise, exhibited greater activation than Shaker and CTAR exercises in both the suprahyoid and infrahyoid muscles in healthy volunteers [40]. However, they reported a lower submental muscle activation during CTAR in patients with dysphagia. They suggested that the reason why this low activation is different from the literature may be that they used a plastic bar instead of a rubber ball. They used a plastic bar instead of a rubber ball in the CTAR exercise and stated that device preference in dysphagic patients could change activation [40]. However, in this study, a rubber ball was used for the CTAR exercise. The reason for the lack of difference between the CTAR and ECC exercises in our study may be that the plastic ball caused an artificial artifact in the measurements taken during the exercise. However, in this study, MVC was measured without contact with sEMG electrodes, and the MVC results of the CTAR exercise group were similar to those of ECC, but superior to those of Shaker. In addition, the results of the ECC exercise group were valid for 60–80% of the 1-RM. Different amounts of muscle activation may occur at different loading rates.

In this study, greater muscle strength was observed in the ECC exercise at the 8^th^ week. This result may be attributed to the structured and controllable parameters of the exercise, which were developed based on volunteers’ competencies. In contrast, the Shaker exercise, in which volunteers carry the weight of their head in the supine position, has some disadvantages for the patient population, particularly in achieving an isometric component [41]. In addition, it is not known how this head weight causes a load on the submental area and the anterior neck region. While isometric CTAR is better tolerated [42], but a ball placed under the head may limit the ROM. Additionally, determining the optimal inflation level of the ball, assessing the resistance of the ball material, and quantifying the amount of loading present challenges. These factors are important for the effective implementation of the CTAR exercise, but they cannot be measured or modified according to the volunteers’ competencies. A possible reason for achieving greater muscle strength in the ECC exercise may be that it was applied according to the 1-RM of the volunteers. This provided a controllable, well-structured loading for each volunteer, and the amount of loading was monitored and updated. Additionally, eccentric training enhances concentric muscle strength and stretch-shortening cycle performance to a greater extent than other methods [10]. The ECC exercise provided the opportunity to build progressive resistance, whereas the other two exercises continued with the same initial loading. Therefore, the volunteers may have continued the strength gain after the 4^th^ week up to the 8^th^ week.

In this study, no differences were found among the three exercises in terms of perceived exertion and pain at the 8^th^ week. Some studies comparing perceived exertion and pain with the same load have reported that concentric exercise causes more exertion and pain during exercise [43,44]. For the isometric component, there are two settings of isometric component comparing Shaker and CTAR exercises: 60 seconds and 10 seconds [17,42]. Because the 10-second contraction only supports the creatine phosphate system, an isometric component setting of 60 s was chosen for this study. Although CTAR is known that CTAR is better tolerated than the Shaker exercise [17], there was no difference among the exercises in this study. In a study investigating the effects of shaker exercise on fatigue, fatigue was found to decrease after six weeks of exercise in healthy elderly individuals [41]. In our study, in support of this finding, there was a significant decrease in perceived exertion level in the Shaker exercise group at 4^th^ week. Although a decrease in perceived exertion and pain levels was observed, the difference was not significant at 8^th^ week. This may be due to the higher physiological capacity of the healthy young population; therefore, the baseline levels were not very high.

In terms of applicability, Shaker and CTAR exercises have certain disadvantages. The Shaker exercise requires the volunteer to lie down, and a suitable environment is required for this [16], whereas the CTAR exercise requires the use of a ball or a plastic bar [38]. ECC exercise may be applied more easily, especially in outpatient follow-ups, as it does not require any equipment or a suitable environment after the amount of resistance is determined. Other eccentric exercises, such as the SMPE and MJOE, present challenges in terms of applicability. The SMPE has been reported to be difficult to understand and perform, even when supported with biofeedback [13]. Similarly, MJOE involves dual-task requirements, as the tongue must be pressed against the palate during isometric contractions. Therefore, it has been reported to be difficult to implement in patients [12]. In our study, all volunteers were successfully oriented to ECC exercises. In addition, eccentric exercise may increase muscle length, and therefore, may pose a risk in elderly patients [45]. However, Koyoma et al. observed positive effects in terms of anterior hyoid displacement with 6-week MJOE, and reported that it was safe in elderly post-stroke patients [12]. In this study, the effects on swallowing kinematics and functions were not examined; however, eccentric exercise did not cause more exertion and pain than other exercises in healthy volunteers, indicating its potential for safe implementation in clinical settings.

The strength of this study is that it is the first to investigate the effect of ECC exercise designed with controllable dose parameters that provide progressive resistance to muscle activation and muscle strength in healthy volunteers. Another strength is the reliability of the MVC measurements. ICC is frequently preferred in rehabilitation research and is an appropriate method for determining the reliability of measurements [46]. During submental sEMG measurements, there may be errors due to electrode placement, artificial artifacts, etc. Therefore, ICC was used to determine the reliability of the results reported in this study. To the best of our knowledge, only one study has investigated the reliability of electrophysiological assessments of the submental muscles [47]. They reported ICC values ranging from moderate to good for the submental muscle activation recorded during different swallowing tasks in young adults. In our study, the intra-rater reliability of MVC was found to be good in the 4th week of the Shaker exercise group. The intra-rater reliability of MVCs recorded at baseline and 8th week of Shaker exercise and at baseline at 4th and 8th weeks of CTAR and ECC exercise groups were found to have good reliability. For inter-rater reliability, the ICC value was found to have excellent reliability for all MVC measurements in the Shaker exercise group. In the CTAR and ECC exercise groups, the inter-rater reliability was found to be reliable for all MVC measurements. This shows that the MVC measurements made in this study have reliability ranging from good to excellent.

### Study limitations and future studies

Although it has been introduced before, this is the first study to determine the efficacy of ECC exercise, for this reason a healthy young adult population was included in the study. Different results may be obtained in different age groups and patients with dysphagia. In addition, as the ECC exercise is an exercise that progresses from maximum mouth opening to closure, the masticatory muscles are also involved in this movement. It is well established that the suprahyoid and masticatory muscles co-contract during functions such as swallowing and chewing. Studies in the field of dentistry have explored how the muscle-specific integrin composition of the masseter muscle changes in cases of cross-bite, and have suggested that orthodontic issues affecting specific teeth may contribute to headaches due to the involvement of masticatory muscles and their connections with the temporomandibular joint [48,49]. While masticatory muscles were not directly assessed in our study, the multifunctional role of muscles in the oral region suggests that future research should consider the potential relationship between exercises targeting the suprahyoid and the masticatory muscles. In our study, the 1-RM method was suitable for home-based exercises. Despite achieving compliance with the amount of resistance in all volunteers, this may not be easily achieved in patients with dysphagia. As a suggestion for future studies, a study can be designed by adjusting the loading parameters over MVC in the participants in face-to-face exercise sessions. Thus, the effects of loading at different MVC percentages on muscle activation and muscle strength can be evaluated. In addition, future research should focus on the efficacy and feasibility of ECC exercise in dysphagic patients, considering factors such as age, medical condition, and compliance with the exercise protocols. In addition, the effects of ECC exercise on muscle strength gain and the amount of muscle activation were investigated in this study. Further studies are needed to determine the effects of ECC exercise on the swallowing function. In addition, this study did not investigate how long the gains obtained from the 8-week exercise program were maintained. The effects of detraining should be investigated in future studies.

## Conclusion

The ECC exercise showed similar effects of MVC to CTAR, while exhibiting greater MVC than the Shaker exercises among healthy volunteers in the 8th week. It was also observed that ECC exercise was more effective in terms of muscle strength gain after 8 weeks of exercise compared to Shaker and CTAR. In addition, these exercises showed similar perceived exertion and pain levels. In terms of clinical relevance, ECC is a well-tolerated, full ROM eccentric exercise model that holds promise as a rehabilitation tool for enhancing suprahyoid muscle activation and muscle strength. Its ability to be adjusted by a person’s abilities, coupled with manageable perceived exertion and pain levels, suggests its potential utility in clinical practice.

## Supporting information

S1 File CONSORT checklist. (DOC)

S2 File Protocol. (PDF)

S3 File Raw data. (XLSX)
