# Supplementary material for: Effects of the eccentric chin closure exercise on submental muscle activation, muscle strength, dysphagia limit, perceived exertion and pain in healthy volunteers: A prospective, randomized parallel group study
Source: PLoS One. 2024 Nov 21;19(11):e0313995. doi: 10.1371/journal.pone.0313995 (PMC11581284; doi:10.1371/journal.pone.0313995)
Supplement: S2 File — (PDF) [file pone.0313995.s002.pdf]

ClinicalTrials.gov Protocol Registration and Results System (PRS) Receipt
Release Date: May 24, 2024

ClinicalTrials.gov ID: NCT05240599

Study Identification

Unique Protocol ID: ECC Exercise

Brief Title: Eccentric Chin Closure Exercise

Official Title: Comparison of the Effects of Three Different Exercises Used in Swallowing Rehabilitation on Suprahyoid Muscle Activation, Muscle Strength, Dysphagia Limit and Perceived Exertion Level

Secondary IDs:

Study Status

Record Verification: May 2024

Overall Status: Completed

Study Start: May 16, 2023 [Actual]

Primary Completion: September 16, 2023 [Actual]

Study Completion: May 1, 2024 [Actual]

Sponsor/Collaborators

Sponsor: Hacettepe University

Responsible Party: Principal Investigator
Investigator: Emre CENGIZ [ecengiz]
Official Title: Research Assistant
Affiliation: Hacettepe University

Collaborators:

Oversight

U.S. FDA-regulated Drug: No

U.S. FDA-regulated Device: Yes

Unapproved/Uncleared Device: No

Pediatric Postmarket Surveillance: No

Product Exported from U.S.: Yes

U.S. FDA IND/IDE: No

Human Subjects Review: Board Status: Approved
Approval Number: 2021/30-13 (KA-21002)
Board Name: Hacettepe University Clinical Research Ethics Committee
Board Affiliation: Hacettepe University

Phone: 0312 305 34 98
Email: kliniketik@hacettepe.edu.tr
Address:

Hacettepe Üniversitesi
Etik Kurulları ve Komisyonları
06100, Sıhhiye/ Ankara

Data Monitoring: No

FDA Regulated Intervention: No

Study Description

Brief Summary: Swallowing is a set of functions that start with the acceptance of food and end with its delivery to the stomach. One of the most important problems associated with swallowing disorders is insufficient airway closure and the risk of aspiration. It is due to the inadequacy of laryngeal elevation that should occur during swallowing. Suprahyoid muscles are the most basic structures responsible for laryngeal elevation. Insufficient activation of the suprahyoid muscles causes insufficient laryngeal elevation.

The suprahyoid muscles consist of a group of muscles located in the anterior region of the neck between the hyoid bone and the mandible. The muscles which forming SH muscles m. digastricus, m. stylohyoideus, m. mylohyoideus and m. geniohyoideus muscles work as a group. SH muscles play a primary role in controlling hyoid bone movement during swallowing due to their relationship with the hyoid bone. It has been reported that the muscle with the highest potential to move the hyoid anteriorly is the geniohyoid muscle, and the mylohyoid muscle has the highest potential to move the hyoid in the superior direction. In addition, in another study, it was stated that since the geniohyoid and mylohyoid muscles have greater structural potential than other SH muscles for anterior and superior displacement of the hyoid, respectively. By understanding the potential for hyoid excursion arising from the structural properties of these muscles, therapists can target specific muscles with exercises designed to promote hyolaryngeal elevation.

Exercises such as Shaker exercise and resistance chin tuck in the literature either directly involve concentric training of the suprahyoid muscles or indirectly aim to gain strength by strengthening the neck flexors. In the light of the available evidence in the literature, eccentric training is also a viable method in swallowing rehabilitation. In eccentric training, the muscle is positioned by shortening its length. Eccentric training can be done by applying resistance to the jaw while the mouth is open and asking the mouth to be closed in a controlled manner against the resistance. In addition, swallowing exercise can be planned by adjusting the mouth opening and placing the SH muscles at the most appropriate angle to generate force. The aim of this study is to compare the effects of these three different exercises on suprahyoid muscle activation, muscle strength, dysphagia limit and perceived exertion level.

Detailed Description: Swallowing is a set of functions that start with the acceptance of food and end with its delivery to the stomach. The oral preparation consists of 4 phases, namely the oral, pharyngeal and esophageal phase. Swallowing disorder (dysphagia) is defined as problems occurring in at least one of the swallowing phases. One of the most important problems associated with swallowing disorders is insufficient airway closure and the risk of aspiration. It is due to the inadequacy of laryngeal elevation that should occur during swallowing. Suprahyoid muscles are the most basic structures responsible for laryngeal elevation. Insufficient activation of the suprahyoid muscles causes insufficient laryngeal elevation.

The suprahyoid (SH) muscles consist of a group of muscles located in the anterior region of the neck between the hyoid bone and the mandible. The muscles which forming SH muscles m. digastricus, m. stylohyoideus, m. mylohyoideus and m. geniohyoideus muscles work as a group. SH muscles play a primary role in controlling hyoid bone movement during swallowing due to their relationship with the hyoid bone. It has been reported that the muscle with the highest potential to move the hyoid anteriorly is the geniohyoid muscle, and the mylohyoid muscle has the highest potential to move the hyoid in the superior direction. In addition, in another study, it was stated that since the geniohyoid and mylohyoid muscles have greater structural potential than other SH muscles for anterior and superior displacement of the hyoid, respectively, these two muscles can be targeted for neuromuscular stimulation preferably. Studies have also shown that exercise can increase motor unit involvement for certain functions. By understanding the potential for hyoid excursion arising from the structural properties of these muscles, therapists can target specific muscles with exercises designed to promote hyolaryngeal elevation.

Interventions to protect the airway in case of swallowing disorder are aimed at increasing the hyolaryngeal elevation. SH muscles provide elevation of the hyolaryngeal complex and also support the opening of the upper esophageal sphincter (UES). The cricopharyngeal muscle, which opens the UES, is opened by the contraction of the SH muscles and the anterior-superior traction of the hyoid and larynx. Insufficient elevation of the hyoid and larynx causes insufficient opening of the UES, resulting in an increase in the amount of pharyngeal residue and the risk of aspiration. Superior hyolaryngeal excursion during swallowing is thought to contribute to airway protection, preventing aspiration. Anterior hyalaryngeal excursion is thought to be associated with the patency of the UES. Exercises such as Shaker exercise and resistance chin tuck in the literature either directly involve concentric training of the suprahyoid muscles or indirectly aim to gain strength by strengthening the neck flexors.

Shaker Exercises were the first exercise developed to increase suprahyoid muscle activation. This exercise, which is characterized by raising the patient's head in the supine position, has been accepted as one of the most basic exercises in dysphagia rehabilitation for many years. In the following years, the Chin Tuck Against Resistance (CTAR) exercise was developed due to the challenging protocol and positional discomfort of the Shaker exercise. In the CTAR exercise, the patient is asked to press a standard size and inflatable ball, which he puts under his chin, towards his sternum. CTAR has become the most commonly used exercise in dysphagia rehabilitation. In the light of the available evidence in the literature, eccentric training is also a viable method in swallowing rehabilitation. In eccentric training, the muscle is positioned by shortening its length. Eccentric training can be done by applying resistance to the jaw while the mouth is open and asking the mouth to be closed in a controlled manner against the resistance. In addition, swallowing exercise can be planned by adjusting the mouth opening and placing the SH muscles at the most appropriate angle to generate force. The aim of this study is to compare the effects of these three different exercises on suprahyoid muscle activation, muscle strength, dysphagia limit and perceived exertion level.

H0 Hypothesis: There is no difference between CTAR, Shaker and Eccentric Chin Closure exercises in terms of suprahyoid muscle activation, suprahyoid muscle strength, dysphagia limit and perceived exertion level in healthy individuals.

H1 Hypothesis: There is a difference between CTAR, Shaker and Eccentric Chin Closure exercises in terms of suprahyoid muscle activation, suprahyoid muscle strength, dysphagia limit and perceived exertion level in healthy individuals.

Conditions

Conditions: Dysphagia

Keywords: Electromyography
Exercise Training
Dysphagia Limit

Study Design

Study Type: Interventional

Primary Purpose: Other

Study Phase: N/A

Interventional Study Model: Parallel Assignment

Number of Arms: 3

Masking: Single (Participant)

Allocation: Randomized

Enrollment: 54 [Actual]

Arms and Interventions

Arms	Assigned Interventions
Experimental: Shaker<br>Shaker exercises consist of isotonic and isometric contractions of the neck flexor muscles. Participants will be asked to lie on their back with their knees straight. Participants will first wait for 60 seconds by lifting their head and looking at their feet. He will repeat the movement three times in total, resting for 60 seconds in between. Then, the participants will raise their heads again, look at the toes, and put their head back on the bed without waiting. By repeating this movement 30 times in total, the exercise program will be completed.<br><br>Individuals will perform this exercise, which consists of isometric components to be repeated 3 times and isotonic components to be performed once, in 3 times per day.	Exercise Training<br>Exercise training will be applied 3 times a day for 8 weeks.
Experimental: Chin Tuck Against Resistance<br>In this exercise, participants have to place an inflatable ball with a diameter of 12 cm between their chin and sternum. This exercise has two subcomponents, isotonic and isometric. In the isometric component, individuals must compress the ball with maximum force between their chin and sternum, hold for 60 seconds, and rest for 60 seconds. One should repeat this isometric component 3 times. In the isotonic parameter, on the other hand, the participants must slowly squeeze the ball between their chin and sternum 30 times with the maximum force they can do. Participants will perform the exercise in an upright sitting position on a back-supported chair.	Exercise Training<br>Exercise training will be applied 3 times a day for 8 weeks.

Arms	Assigned Interventions
Individuals will perform this exercise, which consists of isometric components to be repeated 3 times and isotonic components repeated 10 times in 3 setsand 3 times per day.	
Experimental: Eccentric Chin Closure<br>This exercise will be performed in the form of closing the chin against the manual resistance to be given from the tip of the mandible, starting from the maximum voluntary mouth opening. In this way, eccentric contraction will be created as the suprahyoid muscles will move from the shortest position to the longest position with resistance. Participants will perform the exercise by maintaining the upright posture in the upright sitting position on the back-supported chair. Volunteers will perform this exercise with 10 repetitions in 3 sets and 3 times per day.	Exercise Training<br>Exercise training will be applied 3 times a day for 8 weeks.

Outcome Measures

Primary Outcome Measure:

1. Muscle Activation
   In our study, dual-channel DELSYS Trigno Duo sensors integrated into the software called DELSYS Trigno Lite System will be used. While measuring voluntary muscle activations in the superficial emg device, the values taken will be recorded in microvolts. For superficial emg recording, the high filter pass will be calculated as 20 Hz, the low filter pass as 2 kHz, and the received signal will be amplified 200 times. The signal transition interval will be set to 20 mV.

   [Time Frame: Change from baseline muscle activation at 4 weeks and 8 weeks]
2. Muscle Strength
   Individuals participating in the study will be asked to sit on their backs in an upright position at 90 degrees. Participants' heads will be fixed in a neutral position to prevent possible cervical flexion movement. The head of the digital dynamometer named Jtech Medical Industries Commander Muscle Testing 7633s, which is compatible with the chin area, will be placed under the chin of the participant and the participant will be asked to open his mouth vigorously for 10 seconds against the resistance. This movement will be repeated 3 times, with a 60-second listening period in between. And the maximum and average values taken will be recorded in Newtons.

   [Time Frame: Change from baseline muscle strength at 4 weeks and 8 weeks]

Secondary Outcome Measure:

3. Dysphagia Limit
   Individuals participating in the study will be asked to drink water in 1 ml, 3 ml, 5 ml, 10 ml, 15 ml, 20 ml, 25 ml and 30 ml glasses, respectively. Electricity and sound signals received during drinking water will be followed. The amount of water that people cannot drink in a single swallow (laryngeal elevation) will be determined as the dysphagia limit of that person. This will be decided if the electrical and sound signals received during swallowing the determined amount of water (simultaneously) occur more than once.

   [Time Frame: Change from baseline dysphagia limit at 4 weeks and 8 weeks]
4. Perceived Exertion Level
   After the participants are divided into groups, they will be evaluated in terms of perceived exertion level and pain at the beginning of the exercises, at the 4th week and at the end of the study. For this, the Borg Scale will be used. This scale was developed by Borg to measure the effort expended during physical exercise. The most widely used tool to measure perceived exertion or exercise intensity is Borg's perceived exertion scale. It is the adaptation of the patient's verbal fatigue level at rest and after exertion to the scale. The scale is between 6 and 20. While 6 patients did not feel any fatigue or strain, 20 corresponded to the highest level of fatigue.

   [Time Frame: Change from baseline perceived exertion level at 4 weeks and 8 weeks]

Eligibility

Minimum Age: 18 Years

Maximum Age: 35 Years

Sex: All

Gender Based: No

Accepts Healthy Volunteers: Yes

Criteria: Inclusion Criteria:

- Being between the ages of 18 - 35,
- Volunteering to participate in the study,
- Getting less than 3 points from the T-EAT-10 (Turkish Eating Assessment Test).

Exclusion Criteria:

- Having disc herniation, mechanical neck pain or any pathology in the cervical region.
- Having a temporamandibular joint problem that may affect joint biomechanics and muscle functions.
- Having any neurological or systemic disease,
- Having undergone head and neck surgery or received radiotherapy.

Dischart Criteria

- Individuals who accepted the study and then stopped participating in the study
- Individuals who did not attend the assessments
- Individuals missing 5 days from the weekly follow-up of exercise sessions.

Contacts/Locations

Central Contact Person: Emre CENGIZ [redacted]

Central Contact Backup:

Study Officials: Emre CENGIZ, MSc, PhD(c)
Study Principal Investigator
Hacettepe University

Locations: **Turkey**
Hacettepe University
Ankara, Turkey
Contact: Özlem ULGER, Prof. [redacted]
Contact: Selen SEREL ARSLAN, Assoc. Prof. [redacted]
Principal Investigator: Emre Cengiz, PhD(c)

IPDSharing

Plan to Share IPD: No

References

Citations: Sonoda N, Tamatsu Y. Observation on the attachment of muscles onto the hyoid bone in human adults. Okajimas Folia Anat Jpn. 2008 Nov;85(3):79-90. doi: 10.2535/ofaj.85.79. PubMed 19227198

Pearson WG Jr, Hindson DF, Langmore SE, Zumwalt AC. Evaluating swallowing muscles essential for hyolaryngeal elevation by using muscle functional magnetic resonance imaging. Int J Radiat Oncol Biol Phys. 2013 Mar 1;85(3):735-40. doi: 10.1016/j.ijrobp.2012.07.2370. Epub 2012 Sep 18. PubMed 22995662

Molfenter SM, Steele CM. Physiological variability in the deglutition literature: hyoid and laryngeal kinematics. Dysphagia. 2011 Mar;26(1):67-74. doi: 10.1007/s00455-010-9309-x. Epub 2010 Oct 7. PubMed 20927634

Ludlow CL, Humbert I, Saxon K, Poletto C, Sonies B, Crujido L. Effects of surface electrical stimulation both at rest and during swallowing in chronic pharyngeal Dysphagia. Dysphagia. 2007 Jan;22(1):1-10. doi: 10.1007/s00455-006-9029-4. PubMed 16718620

Matsuo K, Palmer JB. Anatomy and physiology of feeding and swallowing: normal and abnormal. Phys Med Rehabil Clin N Am. 2008 Nov;19(4):691-707, vii. doi: 10.1016/j.pmr.2008.06.001. PubMed 18940636

Pearson WG Jr, Langmore SE, Zumwalt AC. Evaluating the structural properties of suprahyoid muscles and their potential for moving the hyoid. Dysphagia. 2011 Dec;26(4):345-51. doi: 10.1007/s00455-010-9315-z. Epub 2010 Nov 11. PubMed 21069388

Burnett TA, Mann EA, Stoklosa JB, Ludlow CL. Self-triggered functional electrical stimulation during swallowing. J Neurophysiol. 2005 Dec;94(6):4011-8. doi: 10.1152/jn.00025.2005. Epub 2005 Aug 17. PubMed 16107520

Robbins J, Butler SG, Daniels SK, Diez Gross R, Langmore S, Lazarus CL, Martin-Harris B, McCabe D, Musson N, Rosenbek J. Swallowing and dysphagia rehabilitation: translating principles of neural plasticity into clinically oriented evidence. J Speech Lang Hear Res. 2008 Feb;51(1):S276-300. doi: 10.1044/1092-4388(2008/021). PubMed 18230851

Sivarao DV, Goyal RK. Functional anatomy and physiology of the upper esophageal sphincter. Am J Med. 2000 Mar 6;108 Suppl 4a:27S-37S. doi: 10.1016/s0002-9343(99)00337-x. PubMed 10718448

Jacob P, Kahrilas PJ, Logemann JA, Shah V, Ha T. Upper esophageal sphincter opening and modulation during swallowing. Gastroenterology. 1989 Dec;97(6):1469-78. doi: 10.1016/0016-5085(89)90391-0. PubMed 2583413

Cook IJ, Dodds WJ, Dantas RO, Massey B, Kern MK, Lang IM, Brasseur JG, Hogan WJ. Opening mechanisms of the human upper esophageal sphincter. Am J Physiol. 1989 Nov;257(5 Pt 1):G748-59. doi: 10.1152/ajpgi.1989.257.5.G748. PubMed 2596608

Steele CM, Bailey GL, Chau T, Molfenter SM, Oshalla M, Waito AA, Zoratto DC. The relationship between hyoid and laryngeal displacement and swallowing impairment. Clin Otolaryngol. 2011 Feb;36(1):30-6. doi: 10.1111/j.1749-4486.2010.02219.x. PubMed 21414151

Kim Y, McCullough GH. Maximum hyoid displacement in normal swallowing. Dysphagia. 2008 Sep;23(3):274-9. doi: 10.1007/s00455-007-9135-y. Epub 2007 Oct 26. PubMed 17962998

Logemann JA, Rademaker A, Pauloski BR, Kelly A, Stangl-McBreen C, Antinoja J, Grande B, Farquharson J, Kern M, Easterling C, Shaker R. A randomized study comparing the Shaker exercise with traditional therapy: a preliminary study. Dysphagia. 2009 Dec;24(4):403-11. doi: 10.1007/s00455-009-9217-0. Epub 2009 May 27. PubMed 19472007

Yoon WL, Khoo JK, Rickard Liow SJ. Chin tuck against resistance (CTAR): new method for enhancing suprahyoid muscle activity using a Shaker-type exercise. Dysphagia. 2014 Apr;29(2):243-8. doi: 10.1007/s00455-013-9502-9. Epub 2013 Dec 15. PubMed 24337867

Easterling C, Grande B, Kern M, Sears K, Shaker R. Attaining and maintaining isometric and isokinetic goals of the Shaker exercise. Dysphagia. 2005 Spring;20(2):133-8. doi: 10.1007/s00455-005-0004-2. PubMed 16172822

Sze WP, Yoon WL, Escoffier N, Rickard Liow SJ. Evaluating the Training Effects of Two Swallowing Rehabilitation Therapies Using Surface Electromyography--Chin Tuck Against Resistance (CTAR) Exercise and the Shaker Exercise. Dysphagia. 2016 Apr;31(2):195-205. doi: 10.1007/s00455-015-9678-2. Epub 2016 Feb 2. PubMed 26837612

Gao J, Zhang HJ. Effects of chin tuck against resistance exercise versus Shaker exercise on dysphagia and psychological state after cerebral infarction. Eur J Phys Rehabil Med. 2017 Jun;53(3):426-432. doi: 10.23736/S1973-9087.16.04346-X. Epub 2016 Nov 10. PubMed 27830923

Friden J. Changes in human skeletal muscle induced by long-term eccentric exercise. Cell Tissue Res. 1984;236(2):365-72. doi: 10.1007/BF00214240. PubMed 6733763

Roig M, O'Brien K, Kirk G, Murray R, McKinnon P, Shadgan B, Reid WD. The effects of eccentric versus concentric resistance training on muscle strength and mass in healthy adults: a systematic review with meta-analysis. Br J Sports Med. 2009 Aug;43(8):556-68. doi: 10.1136/bjsm.2008.051417. Epub 2008 Nov 3. PubMed 18981046

Kilinc HE, Arslan SS, Demir N, Karaduman A. The Effects of Different Exercise Trainings on Suprahyoid Muscle Activation, Tongue Pressure Force and Dysphagia Limit in Healthy Subjects. Dysphagia. 2020 Aug;35(4):717-724. doi: 10.1007/s00455-019-10079-w. Epub 2019 Nov 25. PubMed 31768618

Demir N, Serel Arslan S, Inal O, Karaduman AA. Reliability and Validity of the Turkish Eating Assessment Tool (T-EAT-10). Dysphagia. 2016 Oct;31(5):644-9. doi: 10.1007/s00455-016-9723-9. Epub 2016 Jul 12. PubMed 27405421

Yoon WL, Khoo JK, Rickard Liow SJ. Chin tuck against resistance (CTAR): new method for enhancing suprahyoid muscle activity using a Shaker-type exercise. Dysphagia. 2014 Apr;29(2):243-8. doi: 10.1007/s00455-013-9502-9. Epub 2013 Dec 15. PubMed 24337867

Ws Coriolano Md, R Belo L, Carneiro D, G Asano A, Al Oliveira PJ, da Silva DM, G Lins O. Swallowing in patients with Parkinson's disease: a surface electromyography study. Dysphagia. 2012 Dec;27(4):550-5. doi: 10.1007/s00455-012-9406-0. Epub 2012 May 27. PubMed 22644084

Aydogdu I, Kiylioglu N, Tarlaci S, Tanriverdi Z, Alpaydin S, Acarer A, Baysal L, Arpaci E, Yuceyar N, Secil Y, Ozdemirkiran T, Ertekin C. Diagnostic value of "dysphagia limit" for neurogenic dysphagia: 17 years of experience in 1278 adults. Clin Neurophysiol. 2015 Mar;126(3):634-43. doi: 10.1016/j.clinph.2014.06.035. Epub 2014 Jul 8. PubMed 25088732

Iida T, Tohara H, Wada S, Nakane A, Sanpei R, Ueda K. Aging decreases the strength of suprahyoid muscles involved in swallowing movements. Tohoku J Exp Med. 2013 Nov;231(3):223-8. doi: 10.1620/tjem.231.223. PubMed 24240663

Skinner JS, Hutsler R, Bergsteinova V, Buskirk ER. The validity and reliability of a rating scale of perceived exertion. Med Sci Sports. 1973 Summer;5(2):94-6. No abstract available. PubMed 4721013

Chen MJ, Fan X, Moe ST. Criterion-related validity of the Borg ratings of perceived exertion scale in healthy individuals: a meta-analysis. J Sports Sci. 2002 Nov;20(11):873-99. doi: 10.1080/026404102320761787. PubMed 12430990

Kane RL, Bershadsky B, Rockwood T, Saleh K, Islam NC. Visual Analog Scale pain reporting was standardized. J Clin Epidemiol. 2005 Jun;58(6):618-23. doi: 10.1016/j.jclinepi.2004.11.017. PubMed 15878476

Links:

Available IPD/Information:
